# Bone metastases of unknown origin: epidemiology and principles of management

**DOI:** 10.1007/s10195-015-0344-0

**Published:** 2015-03-01

**Authors:** Andrea Piccioli, Giulio Maccauro, Maria Silvia Spinelli, Roberto Biagini, Barbara Rossi

**Affiliations:** 1 Oncologic Center, “Palazzo Baleani”, Teaching Hospital Policlinico Umberto I, Rome, Italy; 2 Department of Geriatrics, Orthopedics and Neurosciences, Agostino Gemelli University Hospital, School of Medicine, Catholic University of the SacredHeart, Rome, Italy; 3 Unit of Oncological Orthopaedics, Regina Elena National Cancer Institute, Rome, Italy

**Keywords:** Bone metastases, Unknown origin, Carcinoma

## Abstract

**Abstract:**

Metastases are the most common malignancies involving bone; breast, prostate, lung and thyroid are the main sites of primary cancer. However, up to 30 % of patients present with bone metastases of unknown origin, where the site of the primary neoplasm cannot be identified at the time of diagnosis despite a thorough history, physical examination, appropriate laboratory testing and modern imaging technology (CT, MRI, PET). Sometimes only extensive histopathological investigations on bone specimens from biopsy can suggest the primary malignancy. At other times, a bone lesion can have such a highly undifferentiated histological appearance that a precise pathological classification on routine hematoxylin–eosin-stained section is not possible. The authors reviewed the relevant literature in an attempt to investigate the epidemiology of the histological primaries finally identified in patients with bone metastases from occult cancer, and a strategy of management and treatment of bone metastases from occult carcinomas is suggested. Lung, liver, pancreas and gastrointestinal tract are common sites for primary occult tumors. Adenocarcinoma is the main histological type, accounting for 70 % of all cases, while undifferentiated cancer accounts for 20 %. Over the past 30 years, lung cancer is the main causative occult primary for bone metastases and has a poor prognosis with an average survival of 4–8 months. Most relevant literature focuses on the need for standardized diagnostic workup, as surgery for bone lesions should be aggressive only when they are solitary and/or the occult primaries have a good prognosis; in these cases, identification of the primary tumor may be important and warrants special diagnostic efforts. However, in most cases, the primary site remains unknown, even after autopsy. Thus, orthopedic surgery has a mainly palliative role in preventing or stabilizing pathological fractures, relieving pain and facilitating the care of the patient in an attempt to provide the most appropriate therapy for the primary tumor as soon as possible.

**Level of evidence:**

5

## Introduction

Metastases are the most common type of malignant tumor involving bone; the skeleton is the third most frequent site for metastatic carcinoma after the lung and liver. Any malignant tumor may metastasize to bone: the most common malignancies are breast in women and prostate in men but secondary lesions from lung cancer have risen in both sexes in the last two decades [1–4]. Skeletal lesions can be the first manifestation of malignancy in 25–30 % of cases [4–6]. In recent years, imaging studies have improved, the use of chest and abdominal computed tomography (CT) is increasing and diagnostic endoscopic techniques have advanced; new tumor markers have been identified, guided percutaneous bone biopsy has gained widespread acceptance, immunohistochemistry and even chromosomal analysis have been developed for studying histological specimens so that the primary malignancy is most often identified at an early stage [1, 4, 5, 7, 8]. However, among patients with bone metastases, 22.6–30 % have no evidence of the primary tumor at presentation [2, 8–12]. In fact, unknown primary malignancy is not a well-defined disease entity. On the one hand, it can be considered as a variety of different malignant and metastatic tumors with an occult source at initial presentation. Thus, the initial medical histories, physical examinations and routine laboratory tests fail to detect the site of the primary neoplasm as it is too small and dormant or it has disappeared [13]. In these cases, the histological findings such as immunohistochemical and other morphological parameters from the bone biopsy can be diagnostic. On the other hand, a bone lesion can have such a highly undifferentiated histological appearance that a precise pathological classification on routine hematoxylin–eosin-stained section is not possible [11, 14, 15].

As a consequence, screening and early diagnosis are impossible by definition. The lack of a detectable primary neoplasm delays staging, treatment is challenging, and prognosis and outcome can be uncertain. In any case, even when the primary cancer is unknown, the patient should always be referred as soon as possible to an oncologist after the diagnosis of bone metastasis has been confirmed at biopsy.

## Epidemiology

Metastasis of unknown primary origin is reported to occur in 3–4 % of all cancer patients and 10–15 % of them present with skeletal localizations [2, 13, 16, 17]. The bone is the third most common site of metastatic cancer of unknown primary origin, after the lymph nodes and the lung [12, 17]. Lung, liver, pancreas and gastrointestinal tract are common sites of primary occult tumors. Adenocarcinoma is the main histological type, accounting for 70 % of all cases, while undifferentiated cancer accounts for 15 % and squamous cell carcinomas 10 % [12, 13]. Occult carcinomas are clinically different from their respective manifest forms: with regard to skeletal involvement, the incidence of bone metastases from pulmonary carcinoma is much lower if the primary is occult (4 %) than if it is known (30–50 %); similarly, bone lesions from occult prostate cancer are three times less common than from a known primary, whereas they are four times more common in cases of occult pancreatic primary [12]. Some unknown primary tumors are treatable, like lymphoma, extragonal germ cell neoplasms and ovarian cancer, but the majority of cases have a short fatal clinical course with very scarce possibilities of employing effective chemotherapy [12, 16, 18].

We reviewed the relevant literature in an attempt to investigate the epidemiology of the histological primaries finally identified in patients with bone metastases from occult cancer (Table 1). Since from the end of the 1980s, lung carcinoma was suggested to be the most commonly causative histotype of metastatic bone disease from occult primaries [2, 5, 11, 19–21]. Rougraff et al. [19] described a retrospective analysis of diagnostic workups in 40 patients: lung cancers accounted for 63 % of the identified primaries. Nottebaert et al. [2] found lung carcinomas to be responsible for 52 % of 51 cases of bone lesions from unknown origin, while they accounted for only 7 % of bone metastases with a diagnosed primary. Moreover, patients with skeletal metastases from occult carcinomas showed a high incidence of spinal metastases, cord compression and pathological fractures and a significantly shorter survival compared to bone lesions secondary to known primaries. Over 10 years later, Shih et al. [11] reported similar demographic data (incidence 30 %, male sex and lung prevalence), intractable pain as the predominant symptom, lytic appearance at radiography and poor prognosis. From an analysis of the Swedish Cancer Registry from 1993 to 2008, Hemminki et al. [13] found that patients with metastases from unknown origin diagnosed in the bone mostly died of lung cancer. Vandecandelarae et al. [1] investigated epidemiological changes from the middle of the last century to recent times: a marked increase in lung cancer was noted in all these patients over the last 40 years, especially among women as an obvious demographic effect of smoking; occult breast and prostate cancer reduced their incidence thanks to advances in diagnosis and treatment at an early stage [1]. Among patients admitted in recent years for bone metastases, different authors surprisingly reported an increased incidence of unidentified primaries despite the improvements in diagnostic examinations, new tumor markers, immunohistochemical methods and guided percutaneous biopsy techniques over a 30-year period [1, 5, 22]. Vandecandelarae et al. [1] compared two series of patients with bone metastases from the same rheumatology department, one extending from 1958 to 1967 and the other from 1989 to 1996. Investigations looking for a primary were negative in 9/34 (27 %) patients in the early series and 36/95 (38 %) patients in the recent series. However, these data may reflect the less effective diagnostic and treatment options available in rheumatological institutes, whereas specialized cancer centers now offer many sophisticated diagnostic procedures and valuable therapeutic protocols that can even be performed on an outpatient basis.

**Table 1:** Review of the literature on case distribution and primaries identified in bone metastases of unknown origin

Authors	BMUO at diagnosis	Identified PC	Main PC	PC in order of frequency	Occult PC
Simon and Karluk [14]	12	6	Kidney 3 (50 %)	Kidney (3), lung (2), others (1)	6 (50 %)
Simon and Bartucci [31]	46	20	Lung 7 (35 %)	Lung (7), kidney (6), breast = prostate (2), ovarian = thyroid = liver (1)	26 (56 %)
Nottebaert et al. [2]	51	33	Lung 17 (51 %)	Lung (17), others (16)	18 (35 %)
Shih et al. [11]	52	28	Lung 9 (32 %)	Lung (9), liver (8), kidney (5), prostate (3), thyroid (2), rectum (1)	24 (46 %)
Rougraff et al. [19]	40	34	Lung 23 (67 %)	Lung (23), kidney (4), breast = colon = liver = bladder (1), others (3)	6 (15 %)
Jacobsen et al. [20]	29	24	Lung 11 (46 %)	Lung (11), prostate (3), breast = lymphomas (2), kidney = ovary = pancreas = stomach = small intestine carcinoid = retroperitoneal rhabdomyosarcoma (1)	5 (17 %)
Katagiri et al. [5]	64	59	Lung 23 (39 %)	Lung (23), prostate (11), breast = liver (5), others (15)	5 (8 %)
Vandecandelaere et al. [1]	129	84	Lung 36 (43 %)	Lung (36), prostate (17), kidney (15), breast (9), stomach (2), bladder = colon = testis = pancreas = liver (1)	45 (35 %)
Destombe et al. [24]	107	94	Lung 37 (39 %)	Lung (37), prostate (26), breast (20), bladder (11)	13 (12 %)
Iizuka et al. [25]	27	26	Myeloma 7 (27 %)	Myeloma (7), lymphoma (3), lung (6), prostate (4), kidney = thyroid = liver = pancreas = stomach = esophagus (1)	1 (4 %)
Hemminki et al. [13]	501	256	Lung 128 (50 %)	Lung (128), urinary (29), prostate (16), breast (14), colon (12), pancreas = gastrointestinal (10), liver (9), biliary system (4), stomach (3), mediastinum (2), ovarian (1), others (18)	203 (40 %)

*BMUO* bone metastases of unknown origin, *PC* primary carcinoma

Thus, detection of bone metastases from occult primaries should raise the suspicion that the lungs are the tissue of origin and the suspicion should be stronger in relatively young patients (60–65 years) [1, 13]. After pulmonary origin, bone metastases from undiagnosed renal clear cell carcinomas have increased to 12 %, more than prostate at 10 %, whereas occult thyroid carcinomas are extremely rare (3 %) [1].

As the spine is the most common site of bone metastases, it is also reported to be the most common site of lesions of unknown origin, followed by the pelvis and long bones; lung and thyroid carcinomas should be strongly suspected at this location [21, 23]. However, spinal malignancy of unknown origin is often derived not only from solid tumors, but also from hematological tumors [1, 21, 24, 25]. In the series reported by Iizuka et al. [25], myeloma was the most common etiology (22 %), followed by lung carcinoma, prostate carcinoma and lymphoma. Serological evaluation for monoclonal gammopathy was very useful in revealing the diagnosis of myeloma in all affected patients.

Acrometastases are extremely unusual (<0.1 %), especially as the first presentation of occult carcinoma, but strongly suggest bronchogenic or gastrointestinal cancer [3, 26, 27].

## Strategy of diagnosis and treatment

In patients affected by bone metastases of unknown origin, one of the most important prognostic and treatment-conditioning factors is the histological type, and therefore biopsy is mandatory in an attempt to detect the primary cancer [6, 28, 29]. Biopsy should be performed in the most accessible osseous or concomitant visceral lesion [19, 20, 24] and should include histochemistry, immunohistology and electron microscopy; thus, the surgeon should obtain sufficient material to enable study with special stains, estrogen receptor activity, and hormonal and tumor markers [30]. Bone biopsy is a key component of the diagnostic strategy and histological confirmation is particularly valuable in patients who have a solitary bone metastasis or unusual radiological features suggesting a myeloma or a sarcoma rather than a carcinoma. Although histological studies rarely identify the exact nature of the primary, they often provide important diagnostic clues: highly suggestive histological patterns may be found in small-cell lung cancer, clear-cell renal cancer, or well-differentiated thyroid carcinoma. Immunohistochemistry helps to determine the nature of the primary, most notably when differentiation is minimal. However, Rougraff et al. [19] reported that biopsy alone was unable to identify the primary site of the malignant tumor in 60 % of cases.

Whole-body bone scintigraphy or positron emission tomography (PET)-CT scan, plain radiographs of painful bones and chest–abdominal–pelvic CT are always recommended when occult carcinoma presents with skeletal location regardless of gender. In men, prostate-specific antigen (PSA) levels should be investigated first. In women, mammography is indicated when appropriate immunohistochemistry confirms breast origin; if the mammogram is non-diagnostic and there is histopathological evidence of breast cancer, breast ultrasound and/or magnetic resonance imaging (MRI) should be suggested [31]. Serum protein electrophoresis should be performed as an initial routine study in patients with incidental spinal metastasis [25]. With regard to skeletal findings, the radiographic appearance of the bone lesions is a valuable clue for suggesting the primary; bone CT and MRI are generally used as complementary techniques to confirm the presence of the metastases and to characterize them [3, 6, 12, 18]. Osteolytic lesions typically result from myeloma, renal cell cancer, gastrointestinal tract cancer and melanoma. Osteoblastic metastases can occur from prostate cancer and bronchial carcinoid. The mixed type of metastasis may be seen with breast, lung and cervical cancer. Other morphological features can aid in assessing the source of malignant neoplasms: for example, an expansive and septated metastasis would strongly suggest primary renal cell cancer or thyroid cancer, while intralesional calcifications would suggest a mucinous tumor [3]. Highly hemorrhagic lesions are mostly related to hypernephroma, thyroid cancer and hepatocellular carcinoma [6]. Serum/urine immunofixation and osteomedullary biopsy are advised in the presence of lytic lesions; PSA and thyroglobulin levels are mostly recommended for osteoblastic metastases [12, 18]. Lung tumors can be detected by modern imaging techniques, including PET-CT scan or high resolution spiral CT. However, the sensitivity is low for tumors smaller than 1 cm [13].

On the basis of histology and/or organ-specific clinical symptoms, further diagnostic workup includes abdominal and pelvic ultrasound, bronchoscopy, gastric and intestinal endoscopy, intravenous urography, laparotomy and further site-specific tumor markers. Due to the overall poor prognosis, too many tests to identify the primary at all costs may be inappropriate. If these investigations fail to reveal the primary site, it is unlikely that it will be identified with further extensive diagnostic procedures, but is then mostly established at autopsy [2, 17, 30–32].

The mean survival of these patients has not changed in the last 30 years, ranging from 3 to 12 months from diagnosis [2, 11, 16, 20, 31]. In general, unfavorable prognostic factors for occult primary tumors are male gender, pathological diagnosis of adenocarcinoma and involvement of multiple organs, besides bone dissemination [12, 31]. In terms of histology of primary cancer, lung adversely influenced survival rate, whereas breast and myeloma are favorable [4, 6, 16, 28, 29]. The lung is reported to be the most common site of occult primary tumor with a poor prognosis of only 3 months, whereas breast and prostate cancer survival is relatively favorable at 15 and 23 months, respectively [12, 13, 16, 18, 29, 31, 32]. Patients with a favorable prognosis include men with blastic bone metastases from occult adenocarcinoma and elevated PSA and patients with a single, small and potentially resectable tumor [12, 18, 31].

**Fig. 1:**
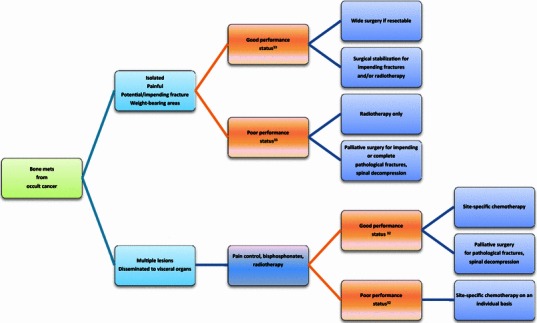
Schematic indications for treatment of patients with bone metastases from occult primary tumor

Probably because of the rarity of occult cancer series and the short survival of bone metastatic patients in general, most of the literature on bone metastases from occult cancer focuses more on the need for a standardized diagnostic flowchart to detect the primary early rather than on a consensus about clinical management when the primary remains undiagnosed [1, 2, 5, 17, 19–21, 25, 32]. Multidisciplinary treatment should attempt to provide local and systemic tumor control in any case; as unknown origin is correlated with a short life expectancy, chemo-radiotherapy and surgery usually have only a palliative role [6, 29]. However, some integrated treatment protocols are potentially curative in a minority of favorable primary diagnoses [12, 18]. In according with current recommendations and guidelines [6, 12, 16, 18, 31, 33], we suggest a flowchart of therapeutic strategy: this approach depends on histological features, patients’ performance status and survival estimation (Fig. 1). The foremost aims of surgery are to preserve the function of the affected bone, to prevent or stabilize pathological fractures, and to relieve pain and facilitate care of the patient while keeping hospitalization as short as possible. Obviously, anatomical site, multiple lesions, visceral involvement and performance status influence surgical options for bone metastases from occult cancer similarly to those of known origin; however, it is especially for bone metastases from unknown primaries that the principle of the more effective and feasible surgical procedures with the lowest rate of complications should be maintained [6, 28, 29, 33]. In the near future, further research on staging examinations, immunohistochemistry, hormone receptor staining and tumor markers may aid in understanding occult tumor characteristics and lead to the most appropriate therapies on an individual basis.

In conclusion, the epidemiology from analysis of the recent literature justifies firstly considering the lungs as the most probable site of primary carcinoma at the onset of bone metastases of undetected origin. The main goal of histology is to identify those primaries for which curative treatment may be available. Efforts should be made to identify the primary and to provide radical treatment in patients who have only one bone metastasis.
